# Using a 2D detector array for meaningful and efficient linear accelerator beam property validations

**DOI:** 10.1120/jacmp.v15i6.4749

**Published:** 2014-11-08

**Authors:** Timothy A. Ritter, Ian Gallagher, Peter L. Roberson

**Affiliations:** ^1^ Department of Radiation Oncology University of Michigan Ann Arbor Michigan USA; ^2^ Department of Radiation Oncology Veterans Affairs Ann Arbor Health Care System Ann Arbor Michigan USA

**Keywords:** linear accelerator, detector array, beam properties, photon characterization

## Abstract

Following linear accelerator commissioning, the qualified medical physicist is responsible for monitoring the machine's ongoing performance, detecting and investigating any changes in beam properties, and assessing the impact of unscheduled repairs. In support of these responsibilities, the authors developed a method of using a 2D ionization chamber array to efficiently test and validate important linear accelerator photon beam properties. A team of three physicists identified critical properties of the accelerator and developed constancy tests that were sensitive to each of the properties. The result was a 14‐field test plan. The test plan includes large and small fields at varying depths, a reduced SSD field at shallow depth for sensitivity to extra focal photon and electron components, and analysis of flatness, symmetry, dose, dose profiles, and dose ratios. Constancy tests were repeated five times over a period of six weeks and used to set upper and lower investigation levels at ±3 SDs. Deliberate variations in output, penumbra, and energy were tested to determine the suitability of the proposed method. Measurements were also performed on a similar, but distinct, machine to assess test sensitivity. The results demonstrated upper and lower investigation levels significantly smaller than the comparable TG‐142 annual recommendations, with the exception of the surrogate used for output calibration, which still fell within the TG‐142 monthly recommendation. Subtle changes in output, beam energy, and penumbra were swiftly identified for further investigation. The test set identified the distinct nature of the second accelerator. The beam properties of two photon energies can be validated in approximately 1.5 hrs using this method. The test suite can be used to evaluate the impact of minor repairs, detect changes in machine performance over time, and supplement other machine quality assurance testing.

PACS numbers: 87.56bd, 87.56Fc

## I. INTRODUCTION

After a linear accelerator is commissioned and placed into service, the qualified medical physicist is responsible for monitoring the machine's ongoing performance and assessing the impact of unscheduled repairs. This quality control (QC) testing is performed as a component of the overall quality assurance (QA) program.^1^, ^2^ QC testing sometimes includes a validation of the beam properties of the accelerator, which we can describe as the spatial, spectral, and dosimetric properties of the output radiation field under the range of expected operating conditions. This type of validation testing typically relies on a subset of the commissioning and acceptance procedure and requires the setup of a scanning water phantom. Facilities routinely perform this scanning as a component of their annual accelerator testing. Major repairs require a similar validation of the beam properties. Even for an experienced physicist, setup and use of the scanning water phantom, with subsequent data analysis, typically takes a full day. Minor repairs usually do not require this level of validation, but sometimes the extent of a repair is unknown or can be deceiving. Several components may be disassembled and reassembled before the offending one is identified and replaced. As a result, the true nature of the required testing can be uncertain. A contingency plan for a thorough and quick validation of machine beam properties is prudent. Consequently, the authors considered a method of using a 2D ionization chamber array, the MatriXX Evolution from IBA Dosimetry (Bartlett, TN), to efficiently validate the constancy of beam properties following minor repairs. This method may also find use as a supplement to the annual machine performance evaluation, or as an “early warning system” for detecting drifts in performance.

## II. MATERIALS AND METHODS

### A. Beam property tests

A Varian TrueBeam (Varian Medical Systems, Palo Alto, CA) operating at 6 MV was the platform used for developing a beam validation method. The mechanical properties of the machine, to include gantry and collimator angular position accuracy, jaw positions, field sizes, and MLC calibrations, are assumed to be validated by other, traditional methods prior to the validation of the beam properties. This approach is consistent with current practices for beam validation using a scanning water phantom.

In order to develop an appropriate beam property test suite, the authors considered a number of factors including conceptual sources of primary and extrafocal radiation, aspects of the control system, and the spectral properties of the fluence exiting the head of the machine.^3^, ^4^ Contributing components were further investigated in the literature, and the findings were used by the authors to generate a minimalist, consensus set of measurements sensitive to the major components. The suite of tests was designed to recognize changes in properties only and not measure absolute values for any parameter, such as focal spot size, penumbra, or energy. This afforded a great advantage in speed and efficiency but, in some cases, may preclude the later decoupling of the properties. The tests were initially designed to be applicable to any planar ionization chamber array. They rely strictly on a comparison of a measured value to a baseline average value generated using a series of reference measurements. A description of the design and purpose of each test field, with letter identifiers A through K, is listed below.

Test field A validates the output constancy of the linear accelerator using a simple measurement of the central axis dose, or a dose surrogate, for a standard test field.

Test field B validates the primary focal spot size, shape, and location using the surrogate of penumbra.^5^, ^6^ Both the X and Y penumbra are evaluated.

Test field C validates the location of the primary focal spot using a test of output at a reduced source‐to‐detector distance. This test serves to ensure the correct inverse square behavior is obeyed.

Test field D checks the component of output dominated by the primary radiation source.^(3)^ It relies on the measured output of a small (less than 3 cm × 3 cm) field.

Test E is sensitive to the primary photon fluence and associated control system. In order to accomplish this evaluation, the two‐dimensional dose distribution for a 20 cm × 20 cm field at a deep location (20 cm) is analyzed. In addition, discrete metrics for energy are assessed. The ratio of the central axis outputs at 20 cm and 10 cm depths forms one metric that is comparable conceptually to a test of tissue‐to‐phantom ratios at these two depths. The flatness at a clinically relevant depth is used as an additional check of energy, as justified by the work of Gao et al.^(7)^

Test F is designed to be sensitive to extrafocal photon and electron fluences and the associated control system. The two‐dimensional dose distribution at a shallow depth (depth of dose maximum or less) for a large field is evaluated.^2^, ^8^ This test is performed at a reduced SSD, which enables the validation of fluence across the largest possible field size.

Test G measures the collimator exchange effect, which is sensitive to extrafocal photon components (e.g. linac head scatter).^9^, ^10^ The exchange effect is tested using the ratio of the output factors for two long, narrow fields with the length greater than five times the width.

Test H measures output linearity which evaluates the control system.^(2)^ The output for a representative field is measured for 5, 50, and 200 monitor units.

Test I assesses the impact of changes in dose rate which evaluates the control system. The output is measured at the highest and lowest available dose rates.

Test J measures changes in output as a function of gantry angle.^(2)^ The output at the four cardinal gantry angles is assessed for a standard field size.

Test K assesses multiple factors using a 1 cm wide MLC‐shaped slit that is delivered using dynamic IMRT delivery.^(11)^ A reference dose value is collected along the central axis and the acquired cross‐plane profile is compared to the baseline. For this test, changes in MLC calibration can impact the effective dosimetric leaf gap and change the results; therefore, a favorable comparison to baseline must be evaluated in combination with all other test results.

### B. Setting up the test fields

The MatriXX Evolution from IBA Dosimetry (Bartlett, TN), a 32 × 32 array of 4.5 mm diameter ion chambers spaced on 7.62 mm centers over a field size of 24 cm × 24 cm, was selected as the 2D measurement array for this project. This device was already in use by our clinic and its performance was characterized and understood by our physics staff. The instrument incorporates automatic temperature and pressure corrections. The interface to the MatriXX device uses the MatriXX IMRT software version 1.7b (IBA Dosimetry). This software acquires the raw charge values from the 1020 ion chambers, applies corrections and calibrations to the data, and can be used to determine a number of key metrics including the calibration correction factor $\left(k_{\text{user}}\right)$, dose values for measured locations (Gy), flatness (%), symmetry (%), and penumbra (mm).

The ionization chamber locations, size, buildup, and backscatter were considered when designing the field dimensions and setup conditions for each constancy test. Plastic Water (Gammex Inc, Middleton, WI) was used both as buildup and backscatter material. Table 1 describes field specifics and setup conditions for each constancy test, the data collected, and the comparisons performed. Jaws were used to shape the fields, with the exception of the swept field test. Each field was delivered at a dose rate of 600 MU/min and for a total of 200 MUs, unless indicated in the table. The specified buildup is in addition to the inherent buildup (3.2 mm) in the MatriXX device. The fields were ordered as indicated in the field label column to minimize the number of changes in buildup and SDD (source‐to‐detector plane distance). In some cases, it was possible to use the same fields to investigate two properties, such as the use of field 10 to test both the collimator exchange effect and X penumbra. Reference values for the metrics, indicated in the final column with a subscript “ref,” indicate the baseline values that the collected data are compared against. The methods of determining these baselines are described in the Materials & Methods section (D) below. In addition to the metrics listed in the table, the planar doses from each field are saved and available for further analysis and investigation.

**Table 1:** A description of the specific field parameters and metrics for each constancy test. SDD refers to the source‐to‐detector (plane) distance. The buildup is in addition to the inherent 3.2 mm buildup in the device.

*Field (s)*	*Test (s)*	*Independent Jaw Positions X1, X2 Y1, Y2*	*Plastic Water Build up (cm)*	*SDD (cm)*	*Gantry / Collimator Angles (degrees)*	*Data Collected (Metric)*	*Comparison(s) Performed*
F1	A	5,5 5,5	5	100	0/0	$k_{\text{user}}$ ${\text{Gy}}_{\text{cax}}(F1)$ ^a^	$1/k_{\text{user}}$ to $1/k_{\text{user},\text{ref}}$
F2, F3, F4	J	5,5 5,5	5	100	270/0 90/0 180/0	${\text{Gy}}_{\text{cax}}(F2)$ ${\text{Gy}}_{\text{cax}}(F3)$ ${\text{Gy}}_{\text{cax}}(F4)$	${\text{Gy}}_{\text{cax}}(F2)$ to ${\text{Gy}}_{\text{cax}}(F{2)}_{\text{ref}}$ ${\text{Gy}}_{\text{cax}}(F3)$ to ${\text{Gy}}_{\text{cax}}(F{3)}_{\text{ref}}$ ${\text{Gy}}_{\text{cax}}(F4)$ to ${\text{Gy}}_{\text{cax}}(F{4)}_{\text{ref}}$
F5, F6	H	5,5 5,5	5	100	0/0	${\text{Gy}}_{\text{cax}}(F5)$ @5 MUs and ${\text{Gy}}_{\text{cax}}(F6)$ @50 MUs	${\text{Gy}}_{\text{cax}}(F5)$ to ${\text{Gy}}_{\text{cax}}(F{5)}_{\text{ref}}$ ${\text{Gy}}_{\text{cax}}(F6)$ to ${\text{Gy}}_{\text{cax}}(F{6)}_{\text{ref}}$
F7	I	5,5 5,5	5	100	0/0	${\text{Gy}}_{\text{cax}}(F7)$ @ 100 MU/min dose rate	${\text{Gy}}_{\text{cax}}(F7)$ to ${\text{Gy}}_{\text{cax}}(F{7)}_{\text{ref}}$
F8	K	6,6 12,12 (Swept 1cm MLC Gap)	5	100	0/0	Profile @ 400MUs, ${\text{Gy}}_{\text{cax}}(F8)$	${\text{Gy}}_{\text{cax}}(F8)$ to ${\text{Gy}}_{\text{cax}}(F{8)}_{\text{ref}}$ X profile comparison to reference
F9	D	0.6, 1.4 0.6, 1.4	5	100	0/0	Gy(F9) ^b^	Gy(F9) to $\text{Gy}(F{9)}_{\text{ref}}$
F10	B	2.3, 2.3 12.5, 12.5	10	100	0/3.8	X jaw penumbra	Xpen^c^ to ${\text{Xpen}}_{\text{ref}}$
F10	G	2.3, 2.3 12.5, 12.5	10	100	0/3.8	${\text{Gy}}_{\text{cax}}(F10)$	[${\text{Gy}}_{\text{cax}}(F10)/{\text{Gy}}_{\text{cax}}(F11)$] to $[{\text{Gy}}_{\text{cax}}(F10)/{\text{Gy}}_{\text{cax}}(F{11)]}_{\text{ref}}$
F11	B	12.5,12.5 2.3,2.3	10	100	0/3.8	Y jaw penumbra	Ypen to ${\text{Ypen}}_{\text{ref}}$
F11	G	12.5,12.5 2.3,2.3	10	100	0/3.8	${\text{Gy}}_{\text{cax}}(F11)$	[${\text{Gy}}_{\text{cax}}(F10)/{\text{Gy}}_{\text{cax}}(F11)$] to $[{\text{Gy}}_{\text{cax}}(F10)/{\text{Gy}}_{\text{cax}}(F{11)]}_{\text{ref}}$
F12	E	10,10 10,10	10	100	0/0	Flatness Symmetry ${\text{Gy}}_{\text{cax}}(F12)$	Flatness(F12) to $\text{Flatness}(F{12)}_{\text{ref}}$ Symmetry(F12) to $\text{Symmetry}(F{12)}_{\text{ref}}$ ${\text{Gy}}_{\text{cax}}(F12)$ to ${\text{Gy}}_{\text{cax}}(F{12)}_{\text{ref}}$
F13	E	10,10 10,10	20	100	0/0	${\text{Gy}}_{\text{cax}}(F13)$ Planar dose	[${\text{Gy}}_{\text{cax}}(F13)/{\text{Gy}}_{\text{cax}}(F12)$] to $[{\text{Gy}}_{\text{cax}}(F13)/{\text{Gy}}_{\text{cax}}(F{12)]}_{\text{ref}}$ Planar dose difference relative to planar $F13_{\text{ref}}$ Profiles relative to $F13_{\text{ref}}$
F14	C	16,16 16,16	1	75	0/0	${\text{Gy}}_{\text{cax}}(F14)$	${\text{Gy}}_{\text{cax}}(F14)$ to ${\text{Gy}}_{\text{cax}}(F{14)}_{\text{ref}}$
F14	F	16,16 16,16	1	75	0/0	Planar dose	Planar dose difference relative reference for F14 Profiles relative reference for F14 Flatness(F14) to $\text{Flatness}(F{14)}_{\text{ref}}$ Symmetry(F14) to $\text{Symmetry}(F{14)}_{\text{ref}}$

^a^ Nomenclature: Reported value in Gy for the central axis chamber, test field F1.

^b^ The reading in Gy from the off‐axis chamber at the center of the small field F9.

^c^ Pen refers to “penumbra” as reported by the Matrixx software.

Table 1 was designed to efficiently convey the specifics of each test field in order to enable the reader to replicate them. Some of the more unfamiliar metrics and terms require further explanation.

The first field, F1, uses the calibration correction factor $k_{\text{user}}$ to assess the output constancy of the machine. The $k_{\text{user}}$ value is utilized by the software to scale the temperature and pressure corrected raw chamber readings and to provide a readout in absolute dose. In this role it mimics an ion chamber calibration factor, only the user is able to acquire the calibration factor by delivering a known dose to the device. $k_{\text{user}}$ is determined in the software as the known dose divided by the average raw values for the four middle chambers of the array.^(12)^ These four chambers, centered around the central axis of the beam, provide an average output over an area of approximately 1.2 cm × 1.2 cm surrounding the CAX. For the first test (A), the $k_{\text{user}}$ factor is used in a reverse role, where the constancy of the calibration factor is used to assess the dose. If the raw chamber responses are precise, then the acquired value of $1/k_{\text{user}}$ can be compared to the reference value and used as an indicator of whether the beam output calibration is constant. Validation that $k_{\text{user}}$ can be used in this role is required.

Fields F2, F3, and F4 assess output constancy as a function of gantry angle. The outputs of the central axis chamber are recorded for gantry angles of 90°, 180°, and 270°. Since a number of comparisons to baseline are performed at a gantry angle of 0°, there is no need to perform an additional constancy check at this angle.

Fields F5 and F6 assess the output constancy for fields delivering 5 MUs and 50 MUs, respectively. When combined with the other fields delivering 200 and up to 400 MUs, the output over a range of clinically significant MUs is validated.

Field F7 assesses the constancy of the output delivered at the lowest clinically available dose rate. Since the other tests use a dose rate of 600 MU/min the goals of Test I are met with the addition of this one field.

Field F8 uses the dynamic delivery of a sliding 1 cm gap formed by the leaves of the MLC. Central axis output and transverse (X) profiles are compared to baseline.

Field F9 uses a 2 cm × 2 cm field centered on a single MatriXX chamber. The output reported by this chamber is compared to the baseline value. A 2 cm × 2 cm field was selected as a compromise size. The smaller the field, the more small changes in jaw calibration and alignment will cause fluctuations in output, while the larger the field, the more extrafocal radiation comes into play.

Fields F10 and F11 are used to assess the collimator exchange effect, as well as the X and Y jaw penumbras. The collimator exchange effect is determined using the central axis output measured for each of these long, narrow fields. The penumbra values reported by the OmniPro IMRT software (Iba Dosimetry) are used to quickly assess penumbra constancy. This value is displayed in the active window of the software when the profile cursor is placed along the field edge. The penumbra is defined in the software as the distance between the 80% and 20% points along the edge of the beam.^(12)^ Given the detector size and spacing, the results were expected to be highly dependent on the positions of the jaws relative to the row of detectors on either side of the field edge. Experimentally this was found to be the case. Deconvolution could be used to determine an absolute penumbra but would require export of the data and a second analytical method, both time‐consuming steps. One method of removing this jaw position dependency would be to always place the jaw edge at the same exact position relative to the detectors, an intractable solution. Instead, these long fields (and therefore jaw edges) were used in combination with a slight collimator rotation. This created a long line of adjacent detector pairs that straddle each jaw edge, with the collimator rotation ensuring that the centering of the jaw edge between detectors was slightly different along the length. The profile cursor location was then “scanned” over the central 20% of the jaw edge to find the local minimum penumbra as reported by the software. This step took only seconds and was intended to reduce the sensitivity to changes in setup. Validation that this technique is repeatable and viable is required.

Fields F12 and F13 are used as comprehensive checks of energy, as described above for Test E. Central axis output, flatness, and symmetry are assessed, while profile and planar dose comparisons to baseline are also performed. Flatness and symmetry are automatically determined over the central 80% of the field width by the software. Flatness is calculated using the relationship:

(1) 100×(Dmax-Dmin)/(Dmax+Dmin)

*Dmax* is the maximum dose measured along the central 80% of the profile, while *Dmin* is the minimum dose over the same region. Symmetry is the maximum difference between the two points equidistant (left and right) from the central axis.

Field F14 is a large field delivered at a reduced SSD. Once again central axis output, flatness, and symmetry are determined, and profiles, as well as planar dose comparisons to baseline, are performed.

### C. Data collection

The Varian TrueBeam used for the experiments is configured with the standard 120 leaf multileaf collimator (MLC), a maximum field size of 40 cm × 40 cm, and the Exact IGRT couch. The 6 MV energy was used for all tests. Before initiating each data collection session, the positioning lasers were verified as accurate to within 1 mm. Two 4 cm thick slabs of plastic water, each piece labeled with a letter and orientation marker, were centered on the couch with the superior edge flush with the couch end. The MatriXX device was carefully centered on this plastic water under the central axis (CAX) of the accelerator with the detector plane set to 100 cm from the source. The detector array was then aligned to the radiation field central axis using an in‐house method. Marks were placed on the solid water at the first data collection to ensure repeatable positioning for subsequent experiments. Data collection was initiated with 5 cm of plastic water centered on top of the MatriXX. As additional slabs were needed, they were always added in the same order and orientation, ensuring that the exact same buildup piece was used in the exact same location from experiment to experiment. Changes in source‐to‐detector distance were accomplished by changing the vertical couch position the required amount, then verifying the shift against the lateral lasers using a steel machinist's rule.

All fields were incorporated into a single plan in the Aria Record and Verify system (Varian Medical Systems). Fields were delivered in QA mode, and data as indicated in Table 2 were collected. The raw data for each acquired field (except for F9, the small field output) were converted to a 0.2 cm grid size along one center line, as described in the OmniPro IMRT 1.7b manual. This conversion effectively up‐sampled the data, with the midpoint of the central pixel placed exactly in the center of the array. For field F9, the raw measured dose for the single chamber at the center of the field was used.

**Table 2:** The results of the constancy tests showing the average and standard deviation for each metric, as well as the upper investigation level (UIL) and lower investigation level (LIL) adopted for each measurement group.

*Metric*	*Avg*	*St Dev* ^a^	*UIL* ^a^	*LIL* ^a^
$1/k_{\text{user},\text{ref}}$	1.317 rdg/cGy	0.56%	+1.7%	−1.7%
${\text{Gy}}_{\text{cax}}(F{2)}_{\text{ref}}$	128.9 cGy	0.12%	+0.81%	−0.81%
${\text{Gy}}_{\text{cax}}(F{3)}_{\text{ref}}$	127.6 cGy	0.17%	+0.81%	−0.81%
${\text{Gy}}_{\text{cax}}(F{4)}_{\text{ref}}$	136.0 cGy	0.13%	+0.81%	−0.81%
${\text{Gy}}_{\text{cax}}(F{5)}_{\text{ref}}$	4.70 cGy	0%	+0.81%	−0.81%
${\text{Gy}}_{\text{cax}}(F{6)}_{\text{ref}}$	47.48 cGy	0.27%	+0.81%	−0.81%
${\text{Gy}}_{\text{cax}}(F{7)}_{\text{ref}}$	189.7 cGy	0.16%	+0.81%	−0.81%
${\text{Gy}}_{\text{cax}}(F{8)}_{\text{ref}}$	36.30 cGy	0.19%	+0.81%	−0.81%
$\text{Gy}(F{9)}_{\text{ref}}$	159.1 cGy	0.18%	+0.81%	−0.81%
${\text{Gy}}_{\text{cax}}(F{14)}_{\text{ref}}$	400.2 cGy	0.25%	+0.81%	−0.81%
$[{\text{Gy}}_{\text{cax}}(F10)/{\text{Gy}}_{\text{cax}}(F{11)]}_{\text{ref}}$	1.013	0.13%	+0.40%	−0.40%
$[{\text{Gy}}_{\text{cax}}(F13)/{\text{Gy}}_{\text{cax}}(F{12)]}_{\text{ref}}$	0.7055	0.09%	+0.40%	−0.40%
${\text{Xpen}}_{\text{ref}}$	0.741 cm	0.30%	+1.1%	−1.1%
${\text{Ypen}}_{\text{ref}}$	0.767 cm	0.36%	+1.1%	−1.1%
$\text{Xflatness}(F{12)}_{\text{ref}}$	2.10%	0.01% (abs)	+0.45% (abs)	−0.45% (abs)
$\text{Yflatness}(F{12)}_{\text{ref}}$	2.36%	0.04% (abs)	+0.45% (abs)	−0.45% (abs)
$\text{Xsymmetry}(F{12)}_{\text{ref}}$	0.22%	0.04% (abs)	+0.45% (abs)	–
$\text{Ysymmetry}(F{12)}_{\text{ref}}$	0.71%	0.11% (abs)	+0.45% (abs)	–
$\text{Xflatness}(F{14)}_{\text{ref}}$	1.75%	0.03% (abs)	+0.45% (abs)	−0.45% (abs)
$\text{Yflatness}(F{14)}_{\text{ref}}$	1.64%	0.03% (abs)	+0.45% (abs)	−0.45% (abs)
$\text{Xsymmetry}(F{14)}_{\text{ref}}$	0.19%	0.03% (abs)	+0.45% (abs)	–
$\text{Ysymmetry}(F{14)}_{\text{ref}}$	0.56%	0.15% (abs)	+0.45% (abs)	–

^a^ % means the number is normalized to the average for the metric, while %(abs) is used for metrics which are already in percent and use an absolute change in percent for the limit.

### D. Setting baselines

Five repeat measurements of the test suite were acquired to set baseline values of all measured parameters. In order to capture realistic variations in results, these measurements were accomplished over a period of six weeks by two different individuals. The mean and standard deviation of the results were calculated and used to determine target values, as well as upper and lower investigation levels. Prior to any data collection, it was anticipated that investigation levels would be set to ±3 SDs to minimize false‐positives (false test failures). For experiments that obey a normal distribution and behave as expected, 99.7% of all results should fall within these limits. This goal can be revisited if the standard deviation is so large that the investigational levels fall significantly outside the comparable TG‐142 recommendations. One of the acquired datasets was also identified as the reference dataset, the standard used to perform future profile and planar dose comparisons. The dataset with the smallest deviation between the measured and mean $k_{\text{user}}$ was selected for this purpose.

### E. Method validation

The utility of an ionization chamber array for assessing photon energy, relative dose, flatness, and symmetry is well established in prior publications.^7^, ^13^ The effect of MLC position on ionization chamber output for a dynamic IMRT field has also been previously reported.^(11)^ Verification that profiles are adequately represented by the array is a prudent step. The ability to detect meaningful changes in focal spot characteristics (through the surrogate of penumbra) and absolute dose calibration (through the surrogate of $k_{\text{user}}$) requires validation. Verification that changes in beam energy are detectable was also performed. In order to test the sensitivity of the method, a different linear accelerator with slightly different beam characteristics was also tested. Use of the method over a period of nine months assessed whether false positives could be expected due to small setup variations or drifts in array response.

Profiles for 20 cm × 20 cm fields were acquired with both the Welhoffer Blue scanning water phantom (IBA Dosimetry) and the MatriXX device underneath plastic water. The Blue phantom was used in combination with CC13 primary and reference ionization chambers, also from IBA Dosimetry. Profiles were obtained at depths of 5 cm and 20 cm and at a 100 cm SSD.

As explained in section B above, the X and Y jaw penumbra values reported by the OmniPro IMRT software for long narrow fields were used to verify penumbra constancy. Tests were accomplished to demonstrate that these reported penumbra results were invariant to jaw position and repeatable. Jaw edges were deliberately shifted from nominal by 2, 1, −1, and −2mm and the penumbra was measured. A second validation test was used to determine if subtle changes in penumbra could be detected. The known change in penumbra with depth was exploited for this test. Penumbra values were measured at depths of 5 cm, 10 cm, 20 cm, and 30 cm in water, using the Wellhofer scanning water phantom described above. Plastic water was then used to achieve the same depth in the MatriXX and changes in penumbra were compared to the scan results.

The ability to detect small changes in output through the surrogate of $k_{\text{user}}$ was validated using manual adjustment of the monitor units (MUs) for a reference 10 cm × 10 cm field. This adjustment of the MUs mimicked a machine calibration error over a range of +10% to −10%. The results were plotted against the inverse of $k_{\text{user}}$, since $k_{\text{user}}$ itself is a correction factor.

A validation experiment tested the impact of changes in photon beam energy. An increase in mean effective energy was accomplished by adding a 6 mm thick (nominal thickness) layer of lead to the beam central axis (CAX) on the accessory tray. This lead covered the entire field and served to harden the beam, but at the same time it also perturbed the photon fluence and generated secondary photons and electrons. Other more realistic methods of simulating changes in energy and fluence were also considered, but they involved altering the accelerator settings. Since the accelerator was in heavy clinical use, these methods were abandoned in favor of the “noninvasive” use of a beam hardening layer. This experiment was only judged to be useful as a way of validating the use of the ratio of two outputs, at 20 cm and 10 cm depths, as a simple discriminator for energy. The change that resulted from the addition of the lead layer was first characterized using a scanning water phantom and an Exradin A12 chamber (Standard Imaging, Middleton, WI). The ratio of the measured percent depth doses at 10 cm (PDD10) and 20 cm (PDD20) depths for both the standard and hardened beam was measured using a 10 cm × 10 cm field. Test fields F12 and F13 were then delivered and measured on the MatriXX using the setup described above, and the ratio ${\text{Gy}}_{\text{cax}}(F13)/{\text{Gy}}_{\text{cax}}(F12)$ was determined.

A second linear accelerator, a Varian 21EX (Varian Medical Systems), is also in use in our department. The head design of this accelerator is similar to the TrueBeam, but there are subtle differences. For example, the ion chamber assembly on the TrueBeam machine is actuated to move when the light field is activated, and there is an antibackscatter filter in the head of the TrueBeam. The properties of the 21EX 6 MV beam are nearly identical to those of the TrueBeam 6 MV beam used for this study, and the machines are calibrated to have identical absolute outputs. The measured percent depth dose at a 10 cm depth for a 10 × 10 field was 66.2% for the 21EX and 66.3% for the TrueBeam. Other beam properties were compared and found to be within 1% for all but the smallest field sizes, a result consistent with the findings of Beyer.^(14)^ Water phantom scan comparisons of this machine and the TrueBeam indicate that they are matched to within TG‐142 tolerances. The suite of tests was run on this alternate machine in order to assess the sensitivity of the method. If the detector array technique is sufficiently sensitive, the subtle differences between the two beams should trigger findings outside the investigation levels.

The test set was run numerous times over a period of nine months following minor repairs and adjustments. The machine underwent beam steering and tuning, jaw calibrations, two output calibration changes of 1% or less, and an ion chamber position calibration during this period. All of the repairs were confirmed by traditional measurements. The MatriXX test suite was then run with the purpose of identifying whether false‐failures were triggered, indicating that the investigation levels were too tight to be clinically useful.

## III. RESULTS & DISCUSSION

Each acquisition of the baseline data took approximately 15 min to set up and no more than 30 min to run once the method was developed. Table 2 displays the results. Upper and lower investigation levels (UILs and LILs, respectively) for each parameter were initially set based on three times the largest observed standard deviation within each of the five logical measurement “families” of $1/k_{\text{user}}$, penumbra, measured dose (in Gy), flatness and symmetry, and ratios of Gy. It is noted that, for practical reasons, the lower investigation level for symmetry will always be 0%. All of the resultant criteria were significantly tighter than the comparable TG‐142 recommended tolerances for monthly or annual testing, with the exception of $1/k_{\text{user}}$ (the analog of output), which was within the monthly recommended level of 2% but exceeded the recommended annual output calibration tolerance of 1%. The very tight investigational levels reflect the advanced digital control capabilities of TrueBeam and demonstrate the benefit of customizing tolerances when possible. The more strict criteria compared to the TG‐142 values make the test suite an effective early warning system for beam property drifts.

The third dataset was identified as the reference and used for profile and planar dose comparisons. The investigation levels for dose (±0.81%) were also used as thresholds for the planar dose difference comparisons which were performed over the central 80% of each field. Comparisons between experiments performed weeks apart by different individuals revealed almost identical profiles that were virtually superimposed when plotted simultaneously. It is noted that, although the comparison of dose planes can provide additional information, profile comparisons were found to be a more practical way of evaluating results.

**Figure 1:**
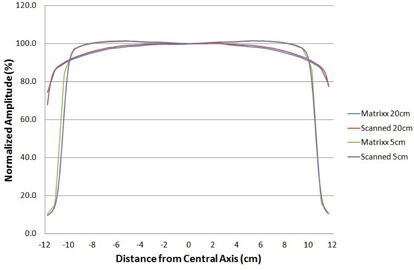
Cross‐plane profiles acquired using a scanning water phantom and the MatriXX. The setup SSD was 100 cm and profiles were acquired at depths of 5 cm and 20 cm. The lateral extent of the area covered by the ionization chambers in the MatriXX is ±12cm.

Comparisons of two profiles measured with the MatriXX and with the scanning water phantom are shown in Fig. 1. Results show a reasonable level of agreement. Since the MatriXX test suite relies on comparisons to baseline measurements acquired on the same array device, it is not critical that the water tank and MatriXX profiles show perfect agreement. Unexpected differences in the magnitude of the horns, misshapen profiles, or severe discontinuities not explained by sampling considerations would warrant further investigation. None were noted.

**Figure 2:**
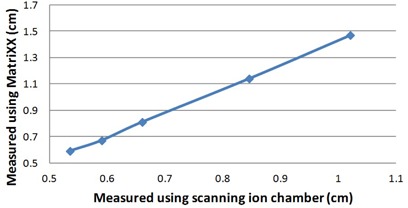
The penumbra reported using the proposed MatriXX‐based method plotted against the same penumbra measured using a scanning ionization chamber.

The penumbra repeatability tests demonstrated a standard deviation of 0.6% over the entire range of jaw positions tested, indicating that using a slight collimator rotation is effective compensation for test‐to‐test variations in jaw position. Figure 2 shows a plot of the penumbra measured with the MatriXX against the penumbra measured with the CC13 ion chamber. An $R^{2}$ value of 0.9995 was calculated for the trend line, indicating a nearly linear relationship. Although the penumbra reported by the OmniPro IMRT software is not a true penumbra, these two results indicate that it serves as a reasonable and quick measure of penumbra constancy. For a more accurate measure of absolute penumbra, the ion chamber readings could be deconvolved with detector size.^(15)^

**Figure 3:**
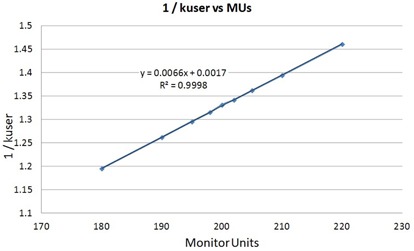
A plot of $1/k_{\text{user}}$ vs. the delivered monitor units. The MatriXX $k_{\text{user}}$ factor was deliberately acquired using the incorrect number of MUs to mimic a machine calibration error. A nearly linear response to the change in MUs is displayed.

Figure 3 displays the plot of $1/k_{\text{user}}$ against the change in monitor units that mimicked a machine calibration error. The response is nearly linear. Changes in $1/k_{\text{user}}$, therefore, appear to be an appropriate surrogate for changes in machine output.

The scanning water phantom tests demonstrated that the addition of lead to the 6 MV beam resulted in a PDD20/PDD10 ratio of 0.587, a 2.3% increase over the value of 0.574 observed for the unhardened beam. The energy change represents a subtle, but clinically significant, shift that should be easily identified by the metric ${\text{Gy}}_{\text{CAX}}(F13)/{\text{Gy}}_{\text{CAX}}(F12)$. The measured ${\text{Gy}}_{\text{CAX}}(F13)/{\text{Gy}}_{\text{CAX}}(F12)$, which is effectively a TPR20/TPR10 ratio, increased by 2.5% to 0.723 with the addition of the lead layer and was well outside the UIL of 0.708. It is noted that profiles were also significantly altered by the lead layer, but the results are not reported because the findings could not be definitively attributed solely to the change in energy.

The results of the 21EX comparison are summarized in Table 3. Profiles and planes appeared nearly identical, but ten of 22 metrics were outside the set investigation levels. Although none of the discrepancies were large, the test suite was sensitive to subtle machine‐to‐machine variations. Three notable differences stand out. The ratio ${\text{Gy}}_{\text{cax}}(F10)/{\text{Gy}}_{\text{cax}}(F11)$ ref is a test designed to be sensitive to the collimator exchange effect. The 21EX result for this metric was approximately 1% higher than the average value reported for the TrueBeam machine. As noted earlier, the monitor chamber is mounted using a different arrangement on the TrueBeam and there is an antibackscatter filter in place. A change in the amount of backscatter into the monitor chamber from the upper jaw could easily account for the measured deviation. The output for field F9 shows a lower value for the 21EX compared to the TrueBeam, pointing to a difference in small field output factors and, possibly, primary focal spot characteristics. The difference in the output for field F8 is likely the result of a number of factors, with the dissimilarity in dosimetric leaf gap offset between the two machines (1.2 mm vs. 1.8 mm) a major contributor.

**Table 3:** The results, other than plane and profile comparisons, of tests performed on a different machine (21EX) with almost identical beam properties. The reference average, upper investigation level, and lower investigation level for each metric are compared to the results obtained for the 21EX. Measured values falling outside the bounds set by the UIL and LIL are labeled as a fail or “F” in column 6.

*Metric*	*Reference Avg*.	*UIL*	*LIL*	*21EX Result*	*Pass (P) or Fail (F)*
$1/k_{\text{user},\text{ref}}$	1.317 rdg/cGy	1.340 rdg/cGy	1.295 rdg/cGy	1.311 rdg/cGy	P
${\text{Gy}}_{\text{cax}}(F{2)}_{\text{ref}}$	128.9 cGy	130.0 cGy	127.9 cGy	130.6 cGy	F
${\text{Gy}}_{\text{cax}}(F{3)}_{\text{ref}}$	127.6 cGy	128.6 cGy	126.6 cGy	128 cGy	P
${\text{Gy}}_{\text{cax}}(F{4)}_{\text{ref}}$	136.0 cGy	137.1 cGy	134.9 cGy	139.3 cGy	F
${\text{Gy}}_{\text{cax}}(F{5)}_{\text{ref}}$	4.70 cGy	4.74 cGy	4.66 cGy	4.8 cGy	F
${\text{Gy}}_{\text{cax}}(F{6)}_{\text{ref}}$	47.48 cGy	47.87 cGy	47.10 cGy	47.4 cGy	P
${\text{Gy}}_{\text{cax}}(F{7)}_{\text{ref}}$	189.7 cGy	191.2 cGy	188.2 cGy	190.9 cGy	P
${\text{Gy}}_{\text{ref}}(F{8)}_{\text{ref}}$	36.30 cGy	36.59 cGy	36.00 cGy	39.0 cGy	F
$\text{Gy}(F{9)}_{\text{ref}}$	159.1 cGy	160.4 cGy	157.9 cGy	156.7 cGy	F
$[{\text{Gy}}_{\text{cax}}(F10)/{\text{Gy}}_{\text{cax}}(F{11)]}_{\text{ref}}$	1.013	1.017	1.009	1.0234	F
${\text{Xpen}}_{\text{ref}}$	0.741 cm	0.749 cm	0.733 cm	0.735 cm	P
${\text{Ypen}}_{\text{ref}}$	0.767 cm	0.775 cm	0.759 cm	0.74 cm	F
$\text{Xflatness}(F{12)}_{\text{ref}}$	2.10%	2.55%	1.65%	2.37%	P
$\text{Yflatness}(F{12)}_{\text{ref}}$	2.36%	2.81%	1.91%	2.34%	P
$\text{Xsymmetry}(F{12)}_{\text{ref}}$	0.22%	0.67%	–	0.93%	F
$\text{Ysymmetry}(F{12)}_{\text{ref}}$	0.71%	1.16%	–	0.57%	P
$[{\text{Gy}}_{\text{cax}}(F13)/{\text{Gy}}_{\text{cax}}(F{12)]}_{\text{ref}}$	0.7055	0.708	0.703	0.7071	P
${\text{Gy}}_{\text{cax}}(F{14)}_{\text{ref}}$	400.2 cGy	403.4 cGy	396.9 cGy	401.4 cGy	P
$\text{Xflatness}(F{14)}_{\text{ref}}$	1.75%	2.2%	1.3%	2.23%	F
$\text{Yflatness}(F{14)}_{\text{ref}}$	1.64%	2.09%	1.19%	2.04%	P
$\text{Xsymmetry}(F{14)}_{\text{ref}}$	0.19%	0.64%	–	0.91%	F
$\text{Ysymmetry}(F{14)}_{\text{ref}}$	0.56%	1.01%	–	0.53%	P

The investigation levels for the proposed method are, in general, significantly tighter than the values used in the report of AAPM Task Group 142.^(2)^ Concern over unnecessary triggers of these levels is, therefore, warranted. Repeat use of the test suite over a period of nine months alleviated these concerns. The experiments were run five times by two different individuals and at different times of the day following machine repairs or adjustments. No failures were noted after any of the minor repairs described above, except for one finding that was marginally outside the investigation level for field F3. This finding was attributed to a slight setup discrepancy, and a repeat measurement confirmed that suspicion. A modest increase in the investigation level for the output metrics, to ±1% instead of the ±0.81%, could be used to further limit unwarranted failures.

In order for this constancy testing to be effective, it is imperative that the performance of the MatriXX be constant over time. IMRT QA experience with the MatriXX device over several years demonstrates this to be the case. User uniformity calibrations can be periodically used to homogenize the response of the chambers, if needed.

If this test suite is used in a clinical scenario it will be used in combination with other QC tests. Vendor recommended and TG‐142–required QA testing would always be performed.^(2)^ Standard daily QA, always performed by the therapists before treatments are delivered, would further validate output. A reference plan from a highly modulated VMAT reference case, or a highly modulated reference IMRT field, could also be tested to validate the delivery of these special modalities.

Most diode or chamber arrays could be used to develop a similar method of machine validation; in fact, a diode array will likely offer a detector size advantage, at a possible cost of additional device calibration measurements. The smaller detector could enable the use of a much smaller reference field for the small field test (test D), further isolating the contribution of the primary radiation source. Almost any detector array would offer a considerable savings of time and complexity compared to setting up a water phantom, although water phantom testing would still be required following major repairs. It is the experience of the authors that setting up a scanning water phantom, performing measurements over a range of setup conditions, and accomplishing meaningful comparisons to baseline values takes more than an 8‐hour day. In contrast, the setup described herein takes approximately 1.5 hrs to acquire the data and perform meaningful analysis for two photon energies.

Future work could involve extending this method to flattening filter‐free photon beams and electrons, and performing additional validation experiments using a “test only” accelerator. Deliberate variations in energy, focal spot size, and other parameters could be introduced to such an accelerator without concern for the potential clinical impact.

## IV. CONCLUSIONS

A simple construct for the radiation contributions from a linear accelerator head was used to develop a test suite capable of efficiently assessing the constancy of beam properties using an ionization chamber array. The suite demonstrated upper and lower investigation levels less than or equal to the comparable TG‐142 recommended annual tolerance values for all properties other than beam output, where a slightly larger but still acceptable investigation level (within the recommended monthly tolerance) was identified. Clinically significant changes in output, beam penumbra, and beam energy were easily detected with this method. This method may be useful for validating beam properties after a minor repair, performing periodic validations of linear accelerator performance constancy (quarterly or semi‐annually), and identifying drifts in machine performance. It may also find application as an adjunct to annual water phantom scanning, since the tight investigation levels used in this method may aid in identifying where a more detailed analysis of the scanning results should be directed.

## Supporting information

Supplementary Material

Supplementary Material

Supplementary Material

Supplementary Material

Supplementary Material

Supplementary Material

Supplementary Material

Supplementary Material

Supplementary Material
