# Supplementary figures and images for: Using a 2D detector array for meaningful and efficient linear accelerator beam property validations
Source: J Appl Clin Med Phys. 2014 Nov 8;15(6):46–58. doi: 10.1120/jacmp.v15i6.4749 (PMC5711127; doi:10.1120/jacmp.v15i6.4749)

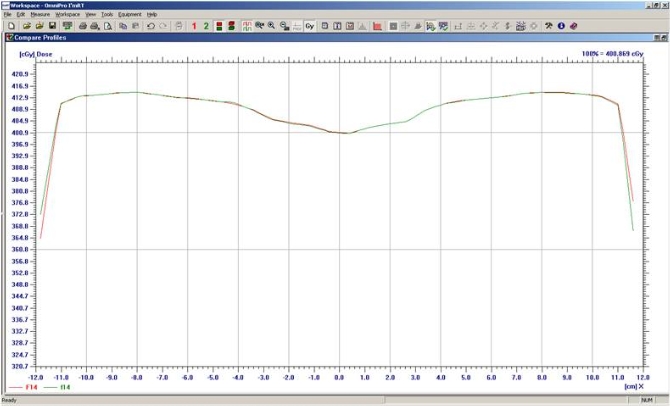

Supplement: Supplementary file 1 — Supplementary Material [file ACM2-15-046-s001.jpg]

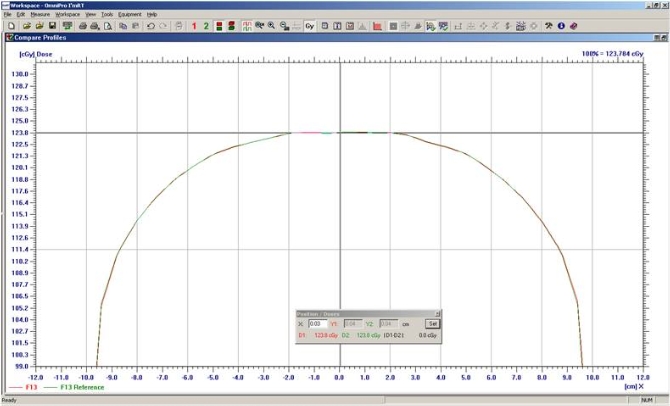

Supplement: Supplementary file 2 — Supplementary Material [file ACM2-15-046-s002.jpg]

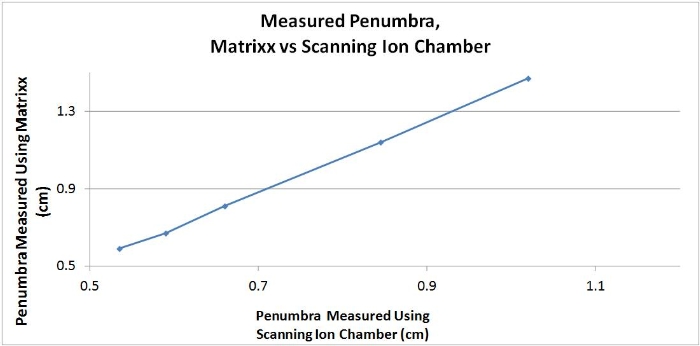

Supplement: Supplementary file 3 — Supplementary Material [file ACM2-15-046-s003.jpg]

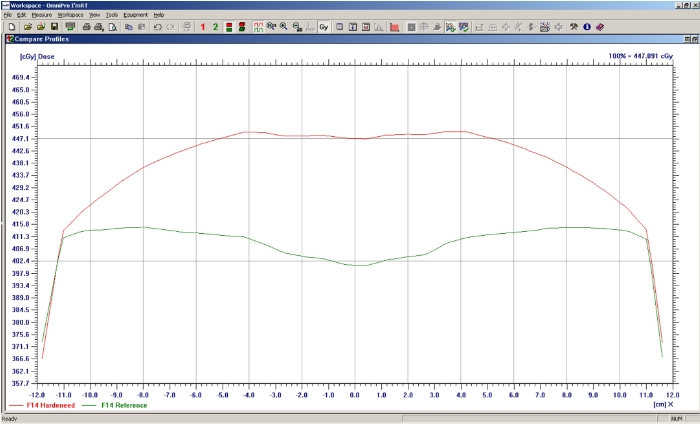

Supplement: Supplementary file 4 — Supplementary Material [file ACM2-15-046-s004.jpg]

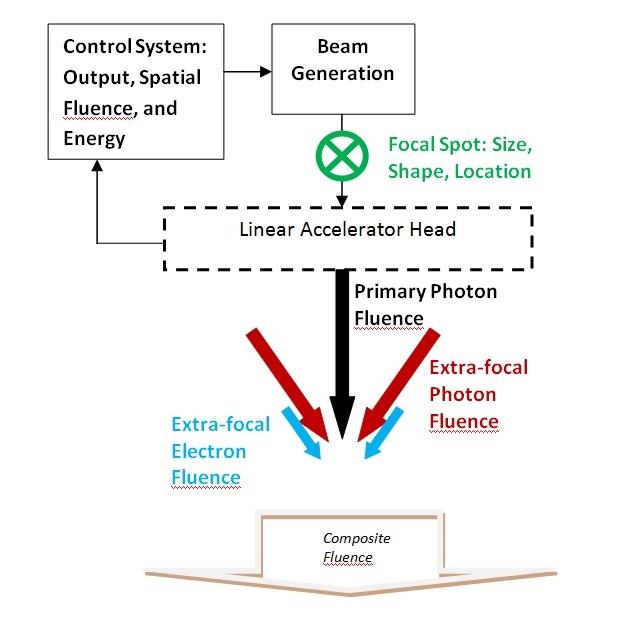

Supplement: Supplementary file 5 — Supplementary Material [file ACM2-15-046-s005.jpg]
